# Comparison of the Effect of Heterologous and Homologous Seminal Plasma on Motility and Chromatin Integrity of Stallion Spermatozoa Selected by Single Layer Centrifugation

**DOI:** 10.1155/2014/325451

**Published:** 2014-08-20

**Authors:** J. M. Morrell, A. Johannisson

**Affiliations:** ^1^Clinical Sciences, Swedish University of Agricultural Sciences (SLU), P.O. Box 7054, 75007 Uppsala, Sweden; ^2^Department of Anatomy, Physiology and Biochemistry, SLU, 75007 Uppsala, Sweden

## Abstract

The effect on sperm motility and chromatin integrity of adding homologous or heterologous equine seminal plasma (SP) to fresh stallion spermatozoa selected by single layer centrifugation (SLC) was studied. No statistical difference in mean progressive motility was seen after adding SP at time 0 h, although there were differences for individual stallions. The proportion of spermatozoa with high velocity was increased compared to untreated SLC-selected spermatozoa (*P* < 0.05), with significant differences between individuals (*P* < 0.01). When the SLC samples were stored for 24 h before adding SP, a significant increase in mean progressive motility was seen for SLC + homologous SP (*P* < 0.01) and for SLC + heterologous SP (*P* < 0.056). Whether homologous SP or heterologous SP had a greater effect on progressive motility depended on the individual. Adding either type of SP caused a significant increase in chromatin damage compared to SLC after storage for 24 h (homologous SP, *P* < 0.05; heterologous SP, *P* < 0.01). These preliminary data showed that storage of SLC-spermatozoa mixed with SP should be avoided because of the risk of increased chromatin damage. If SP is to be added to take advantage of a transient increase in progressive motility for a particular individual stallion, different combinations of SP and spermatozoa should be tested first to optimize the effect.

## 1. Introduction

The effect of seminal plasma (SP), which is the noncellular component of semen, on spermatozoa is the subject of much discussion. The amount of SP may be reduced when preparing semen doses for artificial insemination (AI) because cooled stallion spermatozoa survive better and show greater motility when the seminal plasma is diluted using semen extender [1]. Furthermore, it is common practice to remove most of the SP prior to freezing [2, 3]. However, components of SP have been identified as being beneficial to fertilization, such as the cysteine-rich secretory proteins (CRISP), which are considered to play important roles in sperm physiology, for example, by preventing premature capacitation (CRISP-1 D form), and in sperm-egg interaction, for example, CRISP-1 E form [4]. Moreover, nonprotein SP constituents such as cholesterol may protect the spermatozoa during* in vitro* storage [5].

Recently developed techniques for selecting robust spermatozoa for AI by colloid centrifugation [6, 7] remove all the seminal plasma from the spermatozoa, removing even the SP proteins coating the sperm surface [8]. It is not known what effect this removal has on either the spermatozoa themselves or on the mare's uterus, although stallion spermatozoa selected by single layer centrifugation (SLC) have been shown to be fertile after AI [9, 10] and to function normally in intracytoplasmic sperm injection (ICSI) [11].

There have been previous reports on the beneficial effect of SP on sperm motility [12–14]. In a recent study in which homologous SP was added back to spermatozoa prepared by SLC, a transient increase in progressive motility (PM) and an increase in the proportion of spermatozoa with higher velocity were observed after the addition of SP [15]. However, on storage of the SP-treated spermatozoa, the effect on PM declined and there was an increase in sperm chromatin damage. Adding SP to untreated 24 h stored SLC samples did not affect progressive motility although it did increase the proportion of spermatozoa with a higher velocity [15].

In previous studies in which SP from a different stallion (heterologous SP) was added to sperm samples prior to freezing, some studies showed an improvement in postthaw motility (e.g., [16]) whilst others showed worse postthaw motility than untreated controls [17]. However, establishing a real effect can be difficult unless all the SP is removed from the sperm sample before a known amount is added back. A small cross-over study involving adding SP from sperm-rich and sperm-poor fractions showed that any effect of SP on sperm motility or viability was lost after 24 h [18, 19]. It is difficult to standardise protocols for handling stallion sperm samples when there appears to be so much variation between stallions [17]. Since no comparisons between homologous and heterologous SP on fresh SLC-prepared sperm samples have been made previously, the objective of the current study was to compare the effect of adding back homologous versus heterologous SP to SP-free (SLC-selected) stallion spermatozoa on sperm motility and chromatin integrity, to facilitate devising semen handling protocols for equine AI.

## 2. Materials and Methods

The stallions (17) were kept at a commercial stud farm (Flyinge AB, Flyinge, Sweden), with semen being collected thrice weekly during the breeding season, using a phantom and an artificial vagina according to standard practice. Gel was removed from the ejaculate using an in-line filter. Aliquots (1 mL) of raw ejaculate were centrifuged at 500 g for 10 min to pellet the spermatozoa and collect the SP supernatant. The latter was stored at 6°C until required later the same day. The remaining ejaculate was extended immediately in warm (35°C) INRA96 (IMV, l' Aigle, France). The sperm concentration was measured using a Nucleocounter SP-100 [14] and adjusted to 100 × 10^6^/mL for SLC as previously described [20, 21]. Briefly, 4.5 mL extended semen was carefully layered on top of 4 mL Androcoll-E in a 12 mL centrifuge tube (Sarstedt, Sweden). The Androcoll-E had previously been equilibrated to room temperature (22°C) before use. After centrifuging at 300 g for 20 min, the supernatant and colloid were removed and the pellet transferred to 3 mL fresh INRA96 at ambient temperature (20°C). The sperm concentration in the resulting sperm suspensions was adjusted to give 50 × 10^6^ spermatozoa/mL in 0475 *μ*L aliquots for the following treatments: (i) SLC control; (ii) addition of 25 *μ*L of homologous SP; (iii) addition of 25 *μ*L of pooled heterologous SP (SP from three stallions). Sperm motility was measured immediately using the Qualisperm motility analyzer [22] and again after 24 h cold storage. The Qualisperm results recorded for this experiment were progressive motility (PM; %), velocity (*μ*m/s), as well as the proportion of progressively motile spermatozoa exhibiting Class A motility (rapid progressive motility, >50 *μ*m/s) and Class B motility (slow progressive motility, 10–50 *μ*m/s). In addition, aliquots (100 *μ*L) of the sperm samples were mixed with an equal volume of Tris-sodium ethylenediaminetetraacetic acid buffer (TNE buffer) and snap frozen in liquid nitrogen for subsequent analysis by the sperm chromatin structure assay (SCSA), as reported previously [23]. Parameters measured in the SCSA are the DNA fragmentation index (%DFI), Mean_DFI (mean fluorescence), and SD-DFI (standard deviation of fluorescence).

In a separate experiment, ejaculates from three stallions (H, K and L) were used to determine the effect of adding heterologous SP from individual stallions (M and N) rather than pooled heterologous SP, as in the previous experiment. All experiments were approved by the local ethical committee on animal experimentation (Uppsala Tingsrätt, Sweden).

*Statistics*. The mean proportions of spermatozoa exhibiting different velocity classes and mean values of %DFI, Mean_DFI, and SD_DFI for different treatments were compared by ANOVA using the statistical package for the Excel Software (Version 2003, Microsoft Corporation, Redmond, WA, USA). In all cases, statistical significance was set at *P* < 0.05.

## 3. Results

Mean progressive motility was not significantly affected by addition of either heterologous or homologous SP at 0 h (Table 1) but there was a trend for higher progressive motility in the samples treated with SP at 0 h and assayed after 24 h storage (homologous *P* < 0.056; heterologous *P* < 0.07). In the samples that were stored for 24 h before addition of SP, progressive motility was higher when homologous SP was added than for SLC alone (*P* < 0.01), with a trend towards significance also for the addition of heterologous SP (*P* < 0.056). There was no statistical difference in mean progressive motility between homologous and heterologous SP. There were significant differences between stallions in all treatment groups (*P* < 0.01).

The mean proportion of spermatozoa with Class A velocity was not significantly affected by addition of either homologous or pooled heterologous SP (Table 1). A significant difference was found at 0 h where the SLC + homologous SP group had fewer spermatozoa with Class B motility than SLC-selected spermatozoa (*P* < 0.05). For SP added after 24 h for class, there was a trend for SLC + homologous SP to have a higher proportion of spermatozoa with Class A velocity than SLC (*P* < 0.058). Adding SP from another stallion produced more chromatin damage than the stallion's own SP (Table 2), with a significant increase in %DFI compared to SLC without SP (SLC versus SLC + homologous SP, *P* < 0.05, SLC versus SLC + heterologous SP, *P* < 0.01). Moreover the Mean_DFI was significantly higher for SLC + pooled heterologous SP than for SLC (*P* < 0.05).

**Figure 1:**
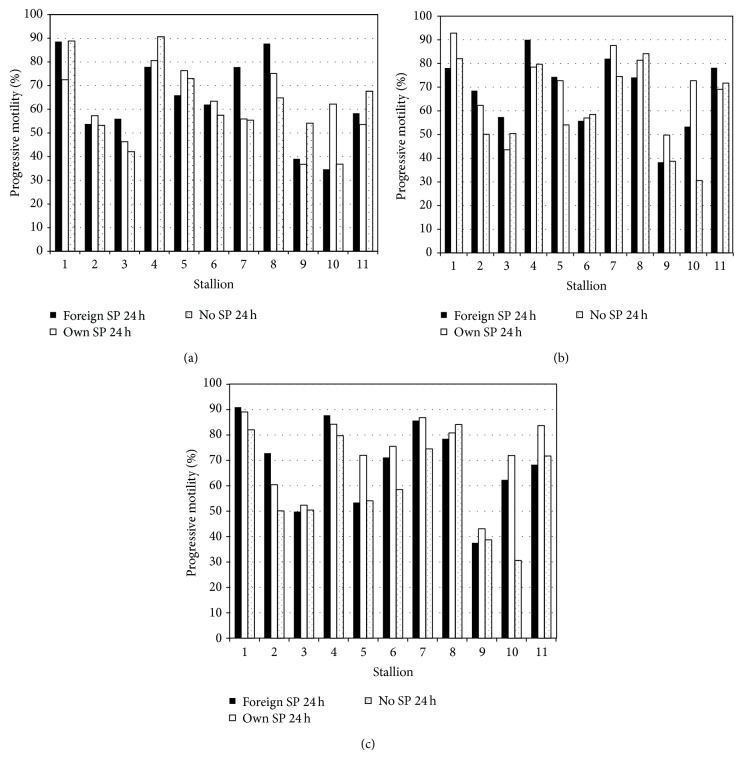
Effect of adding foreign seminal plasma, own seminal plasma, or no seminal plasma to sperm samples prepared by single layer centrifugation on sperm motility; (a) semnal plasma added at 0 h after SLC; (b) 24 h after addition of seminal plasma; (c) seminal plasma added at 24 h after SLC. Note: black bars = foreign seminal plasma, white bars = own seminal plasma, and dotted bars = no seminal plasma.

There was considerable variation between stallions in the response to addition of pooled heterologous SP to fresh SLC-selected samples (Figure 1). For three stallions, PM was the highest in the SLC samples than in SP-treated samples. For six of the 11 stallions, heterologous SP induced a greater increase in PM than homologous SP, but for the remaining five stallions homologous SP caused a greater increase in PM than heterologous SP. In the treated samples after 24 h cold storage, PM was again the highest for pooled heterologous SP for five stallions. However, in the remaining stallions, the highest PM was seen in SLC samples for three stallions and for homologous SP for the other three stallions, although the ranking was not the same as at 0 h. Addition of SP to SLC-samples that had been stored for 24 h after SLC caused a significant increase in PM for ten stallions and a decrease in PM in one stallion (stallion H). Homologous SP caused a greater increase in PM than pooled heterologous SP in seven ejaculates.

The addition of SP from one stallion to SLC-sperm samples from several different stallions produced variable results. For stallion H, own seminal plasma was better than heterologous seminal plasma from stallion M but not from stallion N. In contrast, for stallion L the opposite effect was seen, with heterologous SP from stallion M being better than homologous SP and heterologous SP from stallion N being worse than homologous SP. For stallion K, homologous SP was better than heterologous SP from both stallions M and N.

## 4. Discussion

The aims of this experiment were to compare the effects of adding homologous SP or heterologous SP to SLC-sperm samples on sperm motility and chromatin integrity. The results showed that the effects varied between stallions. For some stallions, PM was higher after the addition of pooled heterologous SP to fresh SLC-samples than homologous SP. However, for other stallions, homologous SP had a greater effect on PM than pooled heterologous SP. When individual heterologous SP was added to stallion semen, the effect on PM was again variable; SP from one stallion could have a beneficial effect compared with homologous SP for one stallion's spermatozoa but a detrimental effect for another stallion's spermatozoa. Adding SP, whether homologous or heterologous, had a detrimental effect on sperm chromatin, as evidenced by increased %DFI and Mean_DFI.

These results confirm our earlier studies on homologous SP, that low concentrations of SP do have a transient beneficial effect on the PM of most SLC-selected sperm samples, but that this effect is lost on storing the treated samples [13–15]. Sperm velocity is also improved by adding SP and this effect is not lost with time. In contrast, storage of SLC-selected sperm samples with SP has a detrimental effect on sperm chromatin [13, 14]. The results are similar to other studies (e.g., [24]) where the presence of SP during cooled storage caused DNA degradation but did not affect sperm motility.

Unfortunately it was not possible to use more than one ejaculate from each stallion in this experiment due to an equipment breakdown after the second day. Therefore, the results of this comparison should be regarded as preliminary. However, they are consistent with anecdotal reports from AI studies in which SP has been added to the semen dose, where condiderable variation is thought to exist between stallions concerning whether homologous or heterologous SP has the greater effect on a given sperm sample (e.g., [16]). The possibility of studying these effects on spermatozoa from which all SP has been removed becomes feasible with SLC, unlike sperm washing where it is not certain that all SP is removed from the spermatozoa or that SP proteins coating the spermatozoa have been removed. A further* in vitro* study is planned in which the effects of adding homologous and heterologous SP to SLC-selected spermatozoa will be compared for a number of stallions and ejaculates. The greater propensity of heterologous SP to cause chromatin damage is an important finding in this study, giving added weight to the argument that if addition of SP is considered to be desirable for sperm function, it should be added immediately before insemination rather than before storage of the sperm dose.

Several previous studies have examined the role of SP proteins on sperm function. SP proteins in ram semen were shown to be associated with sperm protection, capacitation, acrosome reaction, and sperm-oocyte interaction [25]. Stallion SP proteins have been linked to freezability of the semen, for example, CRISP-3 and HSP-2 [26], and IGF-1 has been shown to affect stallion sperm motility [27]. CRISP3 suppresses binding of polymorphonuclear neutrophils to stallion spermatozoa, suggesting that it has a role in regulating sperm elimination from the female reproductive tract [28]. Although SP proteins have been identified as being associated with fertility, observations of their effects on spermatozoa are sometimes contradictory and in many cases their mechanism of action remains to be elucidated. Novak et al. [29] observed that clusterin, kallikrein-1E2, SP1, and SP2 were negatively related to stallion fertility. In agreement with these results, clusterin was negatively linked to human sperm motility and positively linked to DNA damage by Zalata et al. [30], although in contrast Salehi et al. reported that clusterin in human semen correlated negatively with DNA fragmentation [31]. Our own studies have indicated that there is a high degree of variability between stallions in relation to the effect of their SP on spermatozoa [32]; the same may be true of other species, which may account for the conflicting reports in the literature. Of particular interest was the observation that the SP of one out of nine randomly chosen stallions did not cause damage to the sperm DNA of other stallions [32], whereas SP from the other eight stallions did cause damage.

In conclusion, the results presented here show that if SP is to be added to fresh SLC samples, it should be added just prior to AI rather than before storage of the sperm dose. Since there is variation between the effects of SP from one stallion on the spermatozoa of other stallions, it is necessary to test the proposed combination ahead of its use in AI to determine which SP would be beneficial for any individual stallion's spermatozoa.

## Figures and Tables

**Table 1:** Changes in the mean proportion of spermatozoa showing progressive motility (all, Class A, and Class B) after SLC and after the addition of own (homologous) or foreign (heterologous) SP to SLC samples (*n* = 11).

Kinematic	SP added 0 h, motility 0 h			SP added 0 h, motility after 24 h storage			SP added after 24 h		
Progressive motility (%)	SLC only, no SP	Own SP	Foreign SP	SLC only, no SP	Own SP	Foreign SP	SLC only, no SP	Own SP	Foreign SP
All (%)	62 ± 17	62 ± 13	64 ± 18	61 ± 18^ab^	70 ± 15^a^	68 ± 15^b^	61 ± 18^cd^	73 ± 15^c^	69 ± 17^d^
Class A (%)	23 ± 14	24 ± 17	27 ± 16	31 ± 17^f^	40 ± 18^f^	29 ± 15	31 ± 17	47 ± 19	41 ± 23
Class B (%)	40 ± 11^e^	37 ± 10^e^	37 ± 13	30 ± 10	29 ± 11	39 ± 8	30 ± 10	26 ± 10	28 ± 13

Same supercript within a row and treatment indicates significance or trend towards significance. ^a^ *P* < 0.056, ^b^ *P* < 0.07, ^c^ *P* < 0.01, ^d^ *P* < 0.056, ^e^ *P* < 0.05, and ^f^ *P* < 0.058.

**Table 2:** Effects of homologous or heterologous seminal plasma on stallion sperm chromatin integrity.

Treatment	Single layer centrifugation	Homologous SP	Heterologous SP
%DFI	6.3 ± 3.7^ab^	10.6 ± 3.4^a^	11.7 ± 4.0^b^
Mean_DFI	292 ± 5.6^a^	295 ± 8.1	300 ± 10.4^a^
SD_DFI	54.5 ± 13.2	57.0 ± 11.0	59.0 ± 11.6

%DFI = DNA fragmentation index, Mean_DFI = mean fluorescence and SD-DFI standard deviation of fluorescence. SP = Seminal plasma. Seminal plasma was added to SLC samples at 0 h followed by storage at 6°C for 24 h before snap-freezing aliquots for SCSA. Same superscript within a row denotes statistical significance: ^a^ *P* < 0.05; ^b^ *P* < 0.01.
